# Spectrum of Various Mosaicism Types According to Female Age: An Analysis of 36,506 Blastocysts Using Preimplantation Genetic Testing for Aneuploidy

**DOI:** 10.3390/biomedicines13102380

**Published:** 2025-09-28

**Authors:** Min Seo Jeon, Min Jee Kim, Nayeon Choi, Jiseon Hong, Rosa Choi, Yebin Jeong, Hyoung-Song Lee, Kyung Ah Lee, Eun Jeong Yu, Inn Soo Kang

**Affiliations:** 1 Department of Reproductive Genetics Research, CHA Biotech, Seoul Station, Seoul 04637, Republic of Korea; minseo0510@gmail.com (M.S.J.); mjkim44@chamc.co.kr (M.J.K.); chlthddl817@gmail.com (N.C.); ghdwltjs99@gmail.com (J.H.); rschoi0714@gmail.com (R.C.); lenka8601@chamc.co.kr (Y.J.); hslee99@chamc.co.kr (H.-S.L.); 2 Department of Biomedical Sciences, College of Life Sciences, CHA University, Seongnam 13488, Republic of Korea; leeka@cha.ac.kr; 3 Department of Obstetrics and Gynecology, CHA University School of Medicine, CHA Fertility Center Seoul Station, Seoul 04637, Republic of Korea; 4 Department of Obstetrics and Gynecology, CHA University School of Medicine, CHA Fertility Center Daegu, Daegu 41936, Republic of Korea; ikang67pgd@gmail.com

**Keywords:** Embryo transfer, Infertility, Mosaicism, PGT-A

## Abstract

**Background/Objectives**: Mosaicism in preimplantation embryos has important implications for embryo selection and reproductive outcomes. This study investigates the age-related frequency of mosaicism, analyzes its subtypes, and evaluates its clinical significance. **Methods**: A total of 36,506 blastocysts were analyzed using next-generation sequencing-based preimplantation genetic testing for aneuploidy between January 2021 and December 2023. The overall frequencies of euploid, aneuploid, mosaic, and no-call embryos were 20%, 56%, 23%, and 1%, respectively. In this study, we propose a new classification. Embryos were classified into two categories: Mosaic-A, referring to embryos identified as mosaic in standard genetic testing reports, and Mosaic-B, which includes both Mosaic-A and aneuploid embryos containing mosaicism. **Results**: The proportion of Mosaic-A embryos significantly decreased with maternal age (31% in women <35 years, 30% at 35–37 years, 23% at 38–40 years, 16% at 41–42 years, and 10% in women >42 years). By contrast, Mosaic-B embryos, which include Mosaic-A and aneuploid embryos with mosaicism, increased with age (46%, 49%, 53%, 56%, and 62% across the same age groups). Notably, as maternal age advanced, low-level complex mosaicism decreased, whereas high-level complex mosaicism significantly increased (*p* < 0.001, chi-square test for trend). Other mosaicism subtypes followed similar trends. These findings suggest that the increase in high-level complex mosaicism resulted from a higher incidence of post-zygotic mitotic errors occurring earlier in development and affecting a larger proportion of cells in older women. **Conclusions**: This study underscores the significance of incorporating a broader classification of mosaicism, including Mosaic-A and B, to enhance understanding of the biological behavior of mosaic embryos according to age and highlights the clinical importance of cryopreserving high-level or complex mosaic embryos for transfer in women of advanced age.

## 1. Introduction

Preimplantation genetic testing for aneuploidy (PGT-A) is recommended for women of advanced maternal age or those experiencing recurrent implantation failure or miscarriages, as stated in professional guidelines from ASRM (American Society of Reproductive Medicine), ESHRE (European Society of Human Reproduction and Embryology), and PGDIS (Preimplantation Genetic Diagnosis International Society [1,2,3]. The use of PGT-A has grown significantly as a means to enhance live birth rates by supporting the transfer of a single embryo [1,4]. To utilize its potential, PGT-A is being used to detect numerical abnormalities in embryos.

In PGT-A, the embryos are classified into three categories: euploid, aneuploid, and mosaic. Mosaicism refers to the presence of two or more cell populations with different chromosomal compositions in a single embryo. It originates from post-zygotic mitotic errors, such as nondisjunction or anaphase lagging, causing chromosomal segregation anomalies among daughter cells [5]. A related study by Greco et al. (2015), along with subsequent reports, has demonstrated that embryos identified as mosaic through PGT-A can result in viable pregnancies and live births; however, they are associated with lower success rates than those of their euploid counterparts [6]. Additionally, a recent prospective study showed that mosaic embryos had implantation and live birth rates comparable to those of euploid embryos, with similar developmental potential [7]. Similarly, multicenter studies reported no significant differences between euploid and mosaic embryo transfers regarding preterm delivery rates, birth weight, or congenital malformation risk [3,8,9,10,11]. Hence, the transfer of mosaic embryos has become common, offering viable options for couples with limited euploid embryos.

In clinical practice, mosaicism is usually classified into high- and low-level categories based on the percentage of abnormal cells in the trophectoderm (TE) biopsy, with thresholds commonly set at 40% or 50% [12]. This classification facilitates decision-making regarding embryo transfer, as low-level mosaicism is associated with better clinical outcomes than high-level mosaicism [13]. Some clinics have adopted a more refined approach by introducing a “moderate” mosaicism category to further stratify the potential of these embryos [14]. Clinical outcomes vary across mosaicism levels. High-level mosaic embryos are associated with reduced implantation rates and high risks of spontaneous abortion compared with those of low-level or moderate mosaic embryos. Such stratification of the mosaicism levels is critical in optimizing embryo selection and improving success rates in assisted reproductive technologies [12].

PGT-A usually includes TE biopsy because it reflects the entire blastocyst. However, challenges remain in accurately interpreting mosaic findings. Variability in biopsy quality, potential technical artifacts, and subjectivity in PGT-A result interpretation can contribute to false or ambiguous mosaic diagnoses [3]. Despite these advancements, a comprehensive and standardized approach to stratifying mosaicism levels remains limited. Previous studies have primarily focused on broad classifications of mosaicism, usually neglecting the nuanced levels of chromosomal abnormalities [15]. In this study, we aim to address this gap by analyzing a substantial dataset of 36,506 embryos. We focused on further refining mosaicism levels and examining the age-related frequencies of distinct mosaicism types in preimplantation embryos. These findings offer valuable insights into the distribution and occurrence of mosaicism relative to maternal age.

## 2. Materials and Methods

### 2.1. Participants and Data Collection

A total of 36,506 blastocysts from 5938 patients who underwent PGT-A between January 2021 and December 2023 were included in this retrospective, multicenter study. The average maternal age was 39.7 years. The analyzed data were retrieved from the following in vitro fertilization clinics and PGT-A laboratories: CHA Biotech, Laboratory of Reproductive Genetics, CHA Fertility Center Seoul Station, Seoul, Republic of Korea; CHA Fertility Center Daegu, Daegu, Republic of Korea; CHA Fertility Center Gangnam, Seoul, Republic of Korea; CHA Fertility Center Bundang, Gyounggi, Republic of Korea; CHA Fertility Center Ilsan, Gyounggi, Republic of Korea. This study was based on the analysis of previously collected, fully anonymized next-generation sequencing (NGS) data. The data were accessed for research purposes on 1 April 2024. All data were fully anonymized prior to access, and no identifiable information was available to the authors at any stage of the analysis.

**Figure 1:**
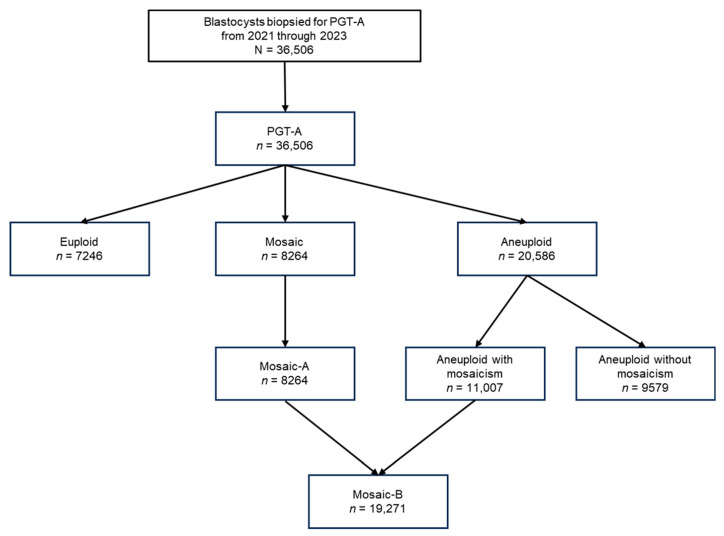
Flowchart of embryo classification based on PGT-A results from 2021 to 2023. A total of 36,506 blastocysts biopsied for PGT-A between 2021 and 2023 were analyzed. Based on chromosomal copy number analysis, embryos were classified as euploid (*n* = 7246), mosaic (*n* = 8264), or aneuploid (*n* = 20,586). Mosaic embryos (hereafter referred to as Mosaic-A) were those designated as mosaic in standard PGT-A reports. Aneuploid embryos were further subdivided into those with mosaic features (*n* = 11,007) and those without (*n* = 9579). A broader category, Mosaic-B, was defined as the union of Mosaic-A embryos and aneuploid embryos exhibiting mosaicism, resulting in a total of 19,271 embryos.

In total, 36,506 blastocysts biopsied for PGT-A between 2021 and 2023 were analyzed. Based on chromosomal copy number analysis, embryos were categorized as euploid (*n* = 7246), mosaic (Mosaic-A, *n* = 8264), or aneuploid (*n* = 20,586) (Figure 1). For data interpretation, mosaicism was defined when copy number values for any genomic region fell within the 20–80% range between monosomy and disomy or between disomy and trisomy. Values <20% were classified as euploid, and >80% as aneuploid.

Following the classification from Viotti’s 2023 study [5], we analyzed the frequency of different mosaicism types (low-level segmental, high-level segmental, low-level one chromosome, low-level two chromosome, low-level complex, high-level one chromosome, high-level two chromosome, and high-level complex) across five SART (Society for Assisted Reproductive Technology) age groups (<35, 35–37, 38–40, 41–42, and >42 years). In this study, low-level and high-level mosaicisms were defined as <50% and ≥50% abnormal cells, respectively.

### 2.2. PGT-A

All embryos in this study underwent blastocyst-stage PGT-A using the VeriSeq NGS platform (Illumina, San Diego, CA, USA). Genomic DNA from lysed cell samples was randomly fragmented and amplified with the SurePlex DNA Amplification System (Illumina) following the manufacturer’s protocol [16]. The amplified products were used to construct DNA libraries according to the VeriSeq PGS workflow, and purified libraries were normalized to ensure equal representation before pooling, denaturation, and sequencing on the MiSeq System (Illumina) with the MiSeq Reagent Kit v.3 (Illumina, San Diego, CA, USA). Sequencing data were analyzed using BlueFuse Multi Software (Illumina, version 4.5). The VeriSeq platform (Illumina, San Diego, CA, USA) used in this study has been validated to reliably detect chromosomal segmental gains and losses ≥20 Mb in size [5].

### 2.3. Definitions of Mosaic Traits

We defined two categories of mosaicism: Mosaic-A and Mosaic-B. Mosaic-A corresponds to the conventional mosaic embryo classification used in PGT-A clinical reports. Mosaic-B is a broader category encompassing both Mosaic-A embryos and aneuploid embryos that exhibit mosaicism. Based on this classification, Mosaic-A embryos accounted for 23% (8264/36,506) of all embryos analyzed, while Mosaic-B included a total of 19,271 embryos: 8264 Mosaic-A embryos and 11,007 aneuploid embryos with mosaic features. Aneuploid embryos without mosaicism totaled 9579 (Figure 1).

Mosaic “level” referred to the inferred percentage of aneuploid cells in a trophectoderm (TE) biopsy specimen. For embryos with two or more mosaic chromosomal regions, the highest level was used for analysis.

Mosaic “type” referred to the nature of the chromosomal abnormality present in the aneuploid cell compartment. Mosaic embryos with exclusively segmental abnormalities were categorized as “single,” “double,” or “complex,” depending on the number of affected segments. Three or more affected segments were considered “complex.” Mosaicism involving a single whole-chromosome (monosomy or trisomy) was considered a “one chromosome” mosaic. Embryos with two whole chromosomes, or one whole chromosome and one segmental region, were classified as “two chromosomes” mosaics. When mosaicism involved more than two whole or segmental chromosomes, the mosaicism type was classified as “complex.”

In this study, we examined the frequency of different mosaicism types and levels across maternal age groups in both Mosaic-A and Mosaic-B categories. This classification framework allowed us to assess not only the visible mosaicism in standard clinical reports, but also the masked mosaicism embedded within aneuploid embryos, thereby offering a more comprehensive view of the biological occurrence of mosaicism in human embryos.

### 2.4. Statistical Analyses

Categorical variables were presented as frequencies with corresponding percentages, and intergroup comparisons were assessed using the chi-square test for trend. Statistical analyses were conducted using GraphPad Prism version 9 (GraphPad Software Inc., La Jolla, CA, USA), with *p*-values < 0.05 considered statistically significant.

All PGT-A analyses in this study were performed at a single laboratory, using standardized protocols and a uniform NGS platform (VeriSeq, Illumina). This centralized testing ensured analytical consistency, minimized inter-laboratory variation, and maintained rigorous quality control across all samples.

## 3. Results

### 3.1. Frequencies of Mosaic-A and -B Across Female Age Groups

**Figure 2:**
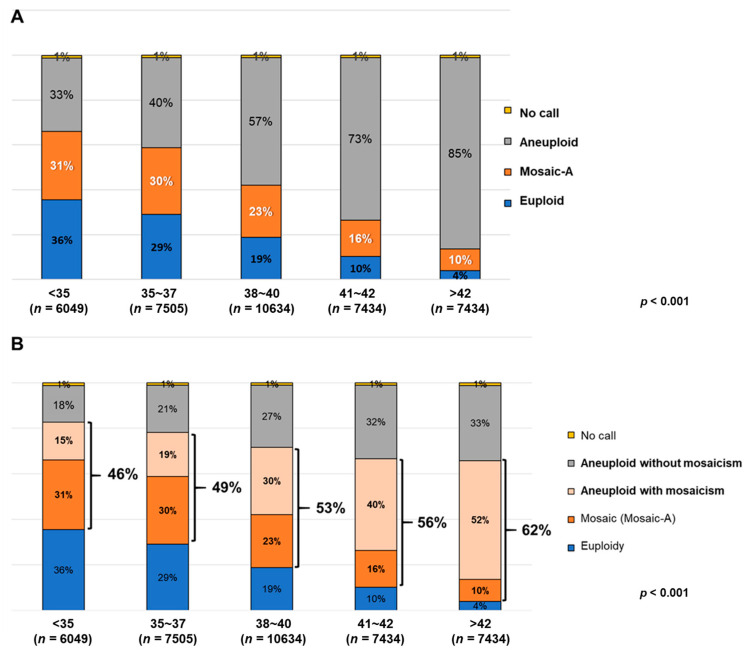
Frequency of mosaic embryo in each category. (**A**) The frequency of the Mosaic-A group (mosaic embryos in PGT-A). (**B**) The frequency of the Mosaic-B group, which comprises mosaic and aneuploid embryos with mosaicism. Significant differences between groups are indicated by *p* < 0.001.

Across 36,506 embryos, the frequency of Mosaic-A embryos significantly decreased with advancing age from 31% in younger age groups to 10% in older groups (Figure 2A). This decline in Mosaic-A may be due to hidden mosaicism within aneuploid embryos, which cannot be identified in standard PGT-A assessments. However, when analyzing the Mosaic-B group, which incorporated mosaicism within aneuploid embryos, an opposite age-related trend was observed (Figure 2B, Table 1). The proportion of Mosaic-B embryos increased from 46% in women under 35 years to 62% in those over 42 years, corresponding to an odds ratio of approximately 1.9. This indicates that the odds of detecting mosaicism within aneuploid embryos were nearly doubled in the oldest maternal age group, underscoring the importance of including Mosaic-B in age-related analyses.

### 3.2. Analysis of the Frequencies of the Mosaicism Subtypes

**Figure 3:**
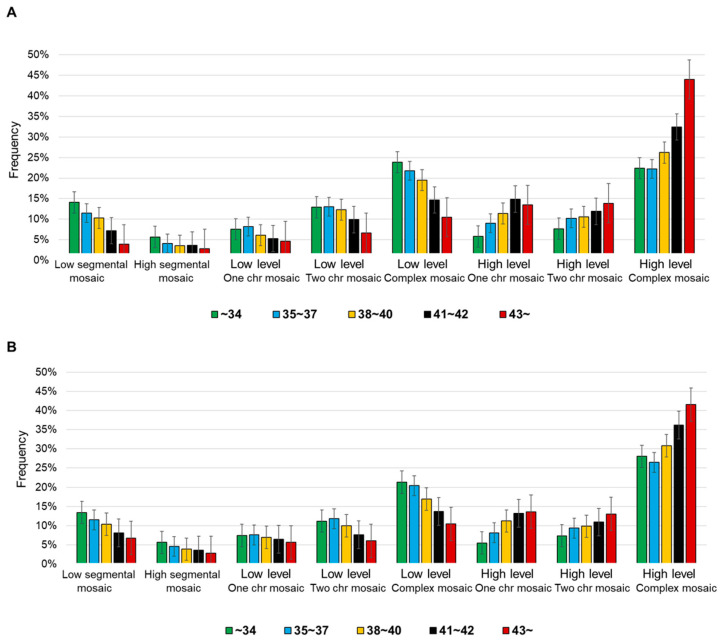
Detailed analysis of the frequencies of Mosaic-A and B according to type and level across female age groups. (**A**) The detailed age-specific frequency of mosaicism in Mosaic-A. (**B**) The detailed age-specific frequency of mosaicism in Mosaic-B. Significant differences between groups are indicated by *p* < 0.001. Percentages are shown for each maternal age category, with error bars indicating standard errors of the mean (SEM).

Further examination of mosaicism subtypes demonstrated a clear age-related shift (Table 2, Figure 3). In the Mosaic-A group, the proportion of high-complex mosaicism increased from 22.4% in women <35 years to 44.0% in women >42 years, while low-complex mosaicism decreased from 23.9% to 10.5% across the same age range. A similar trend was observed in the Mosaic-B group, where high-complex mosaicism rose from 28.0% (<35 years) to 41.6% (>42 years), and low-complex mosaicism decreased from 21.3% to 10.4%**.** These findings indicate that high-level mosaicism, defined by a greater proportion of abnormal cells, became increasingly prevalent with advancing age, whereas low-level mosaicism progressively declined (Figure 2). A chi-square test for trends was also conducted between low- and high-level mosaicism categories, which showed a statistically significant difference (*p* < 0.001).

A more detailed analysis of chromosomal configurations revealed that non-complex mosaicism (including segmental, single-, and two-chromosome mosaics) consistently declined with maternal age, while complex mosaicism became more frequent (Table 2, Figure 3). In the Mosaic-B group, non-complex mosaicism decreased from approximately 37% in women <35 years (13.4% low-segmental, 5.6% high-segmental, 7.4% one-chromosome, 11.2% two-chromosome) to about 21% in women >42 years (6.8%, 2.9%, 5.6%, 6.0%, respectively). Conversely, high-complex mosaicism increased from 28.0% to 41.6%. Mosaic-A embryos exhibited a parallel trend, with non-complex mosaicism declining from 47% to 18%, and high-complex mosaicism nearly doubling from 22.4% to 44.0%. Collectively, these results indicate that maternal aging is associated with a pronounced shift away from simpler, non-complex abnormalities toward increasingly complex chromosomal errors.

Similarly, logistic regression quantifying the odds of high-complex versus low-segmental mosaicism across maternal age groups (reference: <35 years) showed a progressive increase with age. In Mosaic-B, odds were 1.43 (95% CI, 1.22–1.67) at 38–40 years, 2.14 (1.80–2.54) at 41–42 years, and 2.93 (2.42–3.55) at >42 years (Supplementary Table S1). Mosaic-A showed a concordant pattern, with odds reaching 1.60 (1.30–1.98), 2.84 (2.15–3.76), and 7.16 (4.32–11.87), respectively (Supplementary Table S1). See Supplementary Table S1 for details of OR and CI values.

### 3.3. Distribution of Single, Double, and Complex Mosaic Chromosomes

A detailed analysis of chromosomal configurations further confirmed these trends (Table 2, Figure 3). In Mosaic-B embryos, the frequency of single-chromosome mosaics decreased from 7.4% (<35 years) to 5.6% (>42 years), and two-chromosome mosaics showed a similar decline (from 11.2% to 6.0%). Conversely, high-complex mosaicism, defined as mosaicism involving three or more chromosomes, markedly increased with age, from 28.0% in the youngest group to 41.6% in the oldest group. Mosaic-A embryos exhibited parallel changes, with complex mosaicism nearly doubling from 22.4% (<35 years) to 44.0% (>42 years). As in Result 3.2, a chi-square test for trends between non-complex and complex mosaicism also demonstrated a highly significant difference (*p* < 0.001). These results highlight that advancing maternal age is strongly associated with a shift from simpler, low-level chromosomal abnormalities toward complex, high-level mosaic patterns.

These findings indicate that maternal aging drives a shift from simpler to more complex mosaic patterns. Importantly, this age-related trend was consistently observed in both Mosaic-A and the newly defined Mosaic-B categories, highlighting the consistency of this shift across different classifications of mosaicism.

## 4. Discussion

In this study, we proposed a new classification, Mosaic-A and Mosaic-B. Mosaic-A represents the typical mosaic embryos noted in PGT-A reports, while Mosaic-B includes Mosaic-A and aneuploid embryos displaying mosaicism, offering a more comprehensive view of mosaicism’s biological presence.

The primary purpose of introducing the Mosaic-A/B framework was not to suggest a new clinical classification system, but rather to provide a more accurate representation of the biological occurrence of mosaicism across maternal age groups. Our data revealed key age-related trends in mosaicism frequency and complexity, with significant implications for reproductive decision-making. As maternal age advances, the availability of clinically viable euploid and low-level mosaic embryos decreases, while high-level complex mosaicism becomes more common [17,18].

To our knowledge, this is the first comprehensive analysis of mosaicism frequency across both typical and aneuploid embryos, revealing a significant increase in mosaicism from 46% in the youngest age group to 62% in the oldest when Mosaic-B embryos were included. Importantly, this pattern suggests that high-level mosaicism is linked to age-associated cellular processes [7]. For example, low-level complex mosaicism, representing less severe chromosomal disruptions, declines with age; however, high-level complex mosaicism, marked by abnormalities across multiple chromosomes, increases sharply, particularly in women aged > 42 years [17]. This shift suggests that chromosomal stability diminishes with age, leading to an increased likelihood of complex abnormalities in older age. These opposing trends indicate the underestimation of mosaicism frequency when only Mosaic-A is considered, emphasizing the importance of including aneuploid mosaics in assessments to better capture the actual biological occurrence of mosaicism.

Collectively, these insights enhance our understanding of mosaicism in human embryos and highlight the need for evidence-based embryo prioritization strategies in clinical practice. Moreover, our results suggest a potential mechanism underlying this age-associated shift toward complex mosaicism. With increasing maternal age, post-zygotic mitotic errors—particularly during early cleavage stages—may occur more frequently, leading to a greater diversity of chromosomal anomalies among cell clones [19,20]. This observation is consistent with previous reports, which indicated that age-related declines in mitotic fidelity, as well as impaired chromosomal correction mechanisms, may exacerbate mosaicism [7,21]. When compared with other large-scale studies, such as those by Viotti et al. and Capalbo et al. [5,7], our results are concordant in showing an age-related increase in mosaicism complexity.

The introduction of the Mosaic-B category in our analysis extends these earlier findings by systematically including mosaicism within aneuploid embryos, thereby providing a more comprehensive perspective. In addition, the specific vulnerability of certain chromosomal regions, such as repetitive or GC-rich areas, could further contribute to the prevalence of mosaicism, particularly in high-level complex cases [7]. These changes may result from compromised mitotic regulation and reduced correction capacity in older oocytes.

Taken together, this study provides clinically relevant insights into the age-related dynamics of mosaicism, particularly in women of advanced maternal age. While previous reports have suggested that aneuploidy increases with age but mosaicism rates remain constant, those analyses did not account for mosaicism embedded within aneuploid embryos. We believe this may have masked an age-related rise in mosaicism. In our study, by distinguishing Mosaic-A and Mosaic-B, we observed that both the overall proportion of mosaic embryos and the frequency of high-level, complex mosaicism increased with maternal age. Importantly, as age advanced, mosaic embryos represented a larger fraction of transferable embryos. This finding suggests that older patients may face greater difficulty obtaining transferable embryos that are euploid or low-level mosaics. In such cases, even high-level, complex mosaic embryos can be transferred as a last resort, underscoring the clinical relevance of our classification.

This study provides a comprehensive analysis of mosaicism frequency in human embryos, considering Mosaic-A (mosaic embryos in PGT-A reports) and Mosaic-B (including mosaicism in aneuploid embryos). Our findings indicate that the total mosaic frequency increased with maternal age, suggesting that conventional analyses focusing solely on Mosaic-A underestimate the actual prevalence of mosaicism. Despite this overall increase, the proportion of Mosaic-A embryos in PGT-A reports declined significantly with age (from 31% to 10%), likely owing to a masking effect when mosaicism coexists within aneuploid embryos and is less detectable under traditional classifications.

We also observed an age-related shift in mosaicism complexity. As maternal age advanced, the proportion of low-level mosaicism decreased, while high-level complex mosaicism became more common. These shifts with age groups were further supported by odds-ratio analyses: compared with <35 years, the odds of high-complex mosaicism (vs low-segmental) increased to nearly 3-fold in Mosaic-B and over 7-fold in Mosaic-A among women >42 years (Supplementary Table S1). This pattern supports the hypothesis that post-zygotic mitotic errors occur earlier and involve more cell clones in older women, leading to more extensive chromosomal abnormalities. These findings emphasize the clinical relevance of mosaic embryos, particularly for older women. Given the increasing complexity of mosaicism with age, the Mosaic-A/B classification may provide a more comprehensive framework for understanding embryo viability and guiding clinical decision-making.

Given the high frequency of complex mosaicism in this population, high-level mosaic embryos may serve as a critical, albeit challenging, resource. Although these embryos are associated with lower pregnancy rates, they may represent the last viable option for achieving pregnancy—particularly in patients of advanced maternal age, for whom no transferable euploid embryos are available. In such cases, cryopreservation of high-level mosaic embryos could allow for more flexible and time-sensitive decision-making, especially when combined with improved prioritization systems for embryo selection.

### Limitations

This study has several limitations that should be acknowledged. First, its retrospective design may introduce selection bias and unmeasured confounding factors. Although all embryos were analyzed in a single laboratory using the same NGS-based platform under standardized protocols, variability related to ovarian stimulation regimens, patient characteristics, or embryo quality cannot be completely excluded [22]. Second, trophectoderm biopsies may not fully represent the chromosomal constitution of the inner cell mass, limiting the generalizability of our findings to the whole embryo. Although related studies have provided supportive evidence, none can fully capture the phenomenon at 100%, underscoring the complexity of mosaicism and the need for cautious interpretation. Third, the absence of clinical outcome data, such as implantation or live birth rates, prevents direct correlations between mosaicism patterns and reproductive potential. Finally, the proposed biological mechanisms—such as increased mitotic errors in older women—remain hypothetical and were not directly measured in this study. Future prospective, multicenter studies incorporating both molecular data and clinical outcomes will be essential to validate and extend these observations.

## 5. Conclusions

In conclusion, this study highlights a progressive, age-related shift toward complex abnormalities in embryos and demonstrates that the Mosaic-A/B classification offers a broader perspective on mosaicism’s biological presence. Although high-level mosaic embryos have reduced implantation potential, their cryopreservation may be considered as a last-resort option under careful patient counseling and with explicit informed consent. Clinicians should also discuss alternative reproductive strategies, such as donor gametes or adoption, to ensure that patients are fully aware of their choices. By placing the use of high-level mosaic embryos within this broader clinical and ethical context, our findings support more nuanced and individualized reproductive planning.

## Figures and Tables

**Table 1:** Distributions of euploid, mosaic, and aneuploid embryos by maternal age based on preimplantation genetic testing for aneuploidy (PGT-A) (*n* = 36,506). Values are presented as % (95% CI). *p*-values were calculated using the chi-square test for trend. CI = confidence interval.

Age group	Total	<35 Years	35-37 Years	38–40 Years	41–42 Years	>42 Years	
No. of Embryos	N = 36,506	*n* = 6049	*n* = 7505	*n* = 10,634	*n* = 7434	*n* = 4884	*p*-Value
Result Type	% (*n*)	% (*n*)	% (*n*)	% (*n*)	% (*n*)	% (*n*)	
Euploid	19.8% (7246)	34.9% (2110) (33.7~36.1%)	29.1% (2181) (28.0~30.1%)	18.8% (1996) (18.0~19.5%)	10.2% (761) (9.5~10.9%)	4.1% (198) (3.5~4.6%)	<0.001
Mosaic (Mosaic-A, B)	22.6% (8264)	31.2% (1890) (30.1~32.4%)	29.8% (2234) (28.7~30.8%)	23.3% (2474) (22.5~24.1%)	16.1% (1200) (15.3~17.0%)	9.5% (466) (8.7~10.4%)	
Aneuploid with mosaicism (Mosaic-B)	30.2% (11,007)	14.5% (877) (13.6~15.4%)	19.3% (1449) (18.4~20.2%)	29.6% (3143) (28.7~30.4%)	40.2% (2989) (39.1~41.3%)	52.2% (2549) (50.8~53.6%)	
Aneuploid without mosaicism	26.2% (9579)	18.2% (1099) (17.2~19.1%)	20.8% (1561) (19.9~21.7%)	27.3% (2905) (18.9~20.5%)	32.3% (2401) (31.2~33.4%)	33.0% (1613) (31.7~34.3%)	
No Call	1.1% (410)	1.2% (73) (0.9~1.5%)	1.1% (80) (0.8~1.3%)	1.1% (116) (0.9~1.3%)	1.1% (83) (0.9~1.4%)	1.2% (58) (0.9~1.5%)	

The proportion of euploid embryos decreased with age, while the frequency of aneuploid and mosaic embryos increased. This pattern indicates that with advancing maternal age, the proportion of mosaic embryos increases among transferable embryos (euploid and mosaic embryos). Mosaic-A frequency declined with age, whereas Mosaic-B, which includes mosaicism in aneuploid embryos, showed an increasing trend. Statistical significance was determined using a chi-square test for trends (*p* < 0.001).

**Table 2:** Breakdown of the mosaicism subtypes in the Mosaic-A and B groups across maternal age categories.

**Mosaic-A**							
**Age Group**	**Total**	**<35 Years**	**35–37 Years**	**38–40 Years**	**41–42 Years**	**>42 Years**	
**No. of Embryos**	***n* = 8264**	***n* = 1890**	***n* = 2234**	***n* = 2474**	***n* = 1200**	***n* = 466**	***p*-Value**
	**% (*n*)**	**% (*n*)**	**% (*n*)**	**% (*n***)	**% (*n*)**	**% (*n*)**	
Low-segmental mosaic	10.7% (881) (10.0~11.3%)	14.1% (266) (12.5~15.6%)	11.5% (257) (10.2~12.8%)	10.3% (254) (9.1~11.5%)	7.2% (86) (5.7~8.6%)	3.9% (18) (2.1~5.6%)	<0.001
High-segmental mosaic	4.2% (346) (3.8~4.6%)	5.7% (107) (4.6~6.7%)	4.1% (92) (3.3~4.9%)	3.6% (90) (2.9~4.4%)	3.7% (44) (2.6~4.7%)	2.8% (13) (1.3~4.3%)	
One low mosaic	6.8% (564) (6.3~7.4%)	7.6% (143) (6.4~8.8%)	8.2% (183) (7.1~9.3%)	6.1% (152) (5.2~7.1%)	5.3% (64) (4.1~6.6%)	4.7% (22) (2.8~6.6%)	
Two low mosaic	12% (990) (11.3~12.7%)	12.9% (244) (11.4~14.4%)	13% (291) (11.6~14.4%)	12.3% (305) (11.0~13.6%)	9.9% (119) (8.2~11.6%)	6.7% (31) (4.4~8.9%)	
One high mosaic	10.1% (834) (9.4~10.7%)	5.8% (110) (4.8~6.9%)	9.0% (201) (7.8~10.2%)	11.4% (281) (10.1~12.6%)	14.9% (179) (12.9~16.9%)	13.5% (63) (10.4~16.6%)	
Two high mosaic	10.2% (842) (9.5~10.8%)	7.7% (145) (6.5~8.9%)	10.2% (227) (8.9~11.4%)	10.6% (262) (9.4~11.8%)	11.9% (143) (10.1~13.7%)	13.9% (65) (10.8~17.1%)	
Low-complex mosaic	19.9% (1647) (19.1~20.8%)	23.9% (452) (22.0~25.8%)	21.8% (488) (20.1~23.6%)	19.5% (482) (17.9~21.0%)	14.7% (176) (12.7~16.7%)	10.5% (49) (7.7~13.3%)	
High-complex mosaic	26.1% (2160) (25.2~27.1%)	22.4% (423) (20.5~24.3%)	22.2% (495) (20.4~23.9%)	26.2% (648) (24.5~27.9%)	32.4% (389) (29.8~35.1%)	44.0% (205) (39.5~48.5%)	
**Mosaic-B**							
**Age Group**	**Total**	**<35 Years**	**35–37 Years**	**38–40 Years**	**41–42 Years**	**>42 Years**	
**No. of Embryos**	***n* = 19,271**	***n* = 2767**	***n* = 3683**	***n* = 5617**	***n* = 4189**	***n* = 3015**	***p*-Value**
	**% (*n*)**	**% (*n*)**	**% (*n*)**	**% (*n*)**	**% (*n*)**	**% (*n*)**	
Low-segmental mosaic	10% (1922) (9.6~10.4%)	13.4% (372) (12.2~14.7%)	11.5% (424) (10.5~12.5%)	10.3% (581) (9.5~11.1%)	8.1% (340) (7.3~8.9%)	6.8% (205) (5.9~7.7%)	<0.001
High-segmental mosaic	4.1% (784) (3.8~4.3%)	5.6% (156) (4.8~6.5%)	4.6% (170) (3.9~5.3%)	3.9% (218) (3.4~4.4%)	3.7% (154) (3.1~4.2%)	2.9% (86) (2.3~3.4%)	
One low mosaic	6.8% (1320) (6.5~7.2%)	7.4% (206) (6.5~8.4%)	7.6% (280) (6.7~8.5%)	7% (392) (6.3~7.6%)	6.5% (272) (5.7~7.2%)	5.6% (170) (4.8~6.5%)	
Two low mosaic	9.4% (1807) (9.0~9.8%)	11.2% (310) (10.0~12.4%)	11.8% (435) (10.8~12.9%)	10% (560) (9.2~10.8%)	7.6% (320) (6.8~8.4%)	6% (182) (5.2~6.9%)	
One high mosaic	10.6% (2048) (10.2~11.1%)	5.5% (153) (4.7~6.4%)	8.2% (301) (7.3~9.1%)	11.2% (630) (10.4~12.0%)	13.2% (553) (12.2~14.2%)	13.6% (411) (12.4~14.9%)	
Two high mosaic	10.1% (1953) (9.7~10.6%)	7.4% (204) (6.4~8.3%)	9.4% (345) (8.4~10.3%)	9.8% (553) (9.1~10.6%)	10.9% (458) (10.0~11.9%)	13.0% (393) (11.8~14.2%)	
Low-complex mosaic	16.5% (3186) (16.0~17.1%)	21.3% (590) (19.8~22.8%)	20.4% (753) (19.1~21.7%)	17.0% (953) (16.0~17.9%)	13.7% (575) (12.7~14.8%)	10.4% (315) (9.4~11.5%)	
High-complex mosaic	32.4% (6251) (31.8~33.1%)	28.0% (776) (26.4~29.7%)	26.5% (975) (25.0~27.9%)	30.8% (1730) (29.6~32.0%)	36.2% (1517) (34.8~37.7%)	41.6% (1253) (39.8~43.3%)	

Values are presented as % (95% CI). *p*-values were calculated using the chi-square test for trends. CI = confidence interval.

## Data Availability

The data presented in this study are not publicly available due to privacy and ethical restrictions. Aggregated anonymized data may be available from the corresponding author on reasonable request.

## Supplementary Materials

The following supporting information can be downloaded at: https://www.mdpi.com/article/10.3390/biomedicines13102380/s1, Table S1: Odds of high-complex vs low-segmental mosaicism across age.

## Abbreviations

The following abbreviations are used in this manuscript:

ASRM	American Society of Reproductive Medicine
ESHRE	European Society of Human Reproduction and Embryology
NGS	Next-Generation Sequencing
PGDIS	Preimplantation Genetic Diagnosis International Society
PGS	Preimplantation Genetic Screening
PGT-A	Preimplantation Genetic Testing for Aneuploidy
TE	Trophectoderm
