# Supplementary material for: Spectrum of Various Mosaicism Types According to Female Age: An Analysis of 36,506 Blastocysts Using Preimplantation Genetic Testing for Aneuploidy
Source: Biomedicines. 2025 Sep 28;13(10):2380. doi: 10.3390/biomedicines13102380 (PMC12562040; doi:10.3390/biomedicines13102380)
Supplement: Supplementary file 1 [file biomedicines-13-02380-s001.zip › biomedicines-3836720-supplementary.pdf]

**Supplementary Table S1. Odds of high-complex vs low-segmental mosaicism across age (ref: <35y)**

| Age group | Mosaic-A OR (95% CI) | p-value | Mosaic-B OR (95% CI) | p-value |
|---|---|---|---|---|
| <35 years | 1.00 (reference) | – | 1.00 (reference) | – |
| 35–37 yrs | **1.21** (0.98–1.50) | $p=0.081$ | **1.10** (0.93–1.30) | $p=0.256$ |
| 38–40 yrs | **1.60** (1.30–1.98) | <0.0001 | **1.43** (1.22–1.67) | $p<0.0001$ |
| 41–42 yrs | **2.84** (2.15–3.76) | <0.0001 | **2.14** (1.80–2.54) | $p<0.0001$ |
| >42 yrs | **7.16** (4.32–11.87) | <0.0001 | **2.93** (2.42–3.55) | $p<0.0001$ |

Logistic regression analysis of maternal age and the odds of high-complex mosaicism compared with low-segmental mosaicism, stratified by Mosaic-A and Mosaic-B classifications. Odds ratios (ORs), 95% confidence intervals (CIs), and $p$-values are shown for each maternal age group (<35 years as the reference). Low-segmental mosaicism was chosen as the reference category because it represents the least severe and most clinically interpretable subtype, thereby serving as a conservative baseline against which the age-related increase in high-complex mosaicism could be quantified.
